# The Council on Pharmacy and Chemistry

**Published:** 1907-06

**Authors:** 


### The Council on Pharmacy and Chemistry.

The *Journal-Record* is in receipt of a communication from one W. A. Puckner, who describes himself as secretary of the Council on Pharmacy and Chemistry of the American Medical Association. The communication contains the information that a reprint has been sent us of the New and Non-Official Remedies which have been published in the *Journal.* A polite invitation for criticism and suggestion for those interested in the work is included. The editor is furthermore addressed as "Mr."

Of this invitation we shall avail ourselves. Inasmuch as the *Journal-Record* (with its progenitors) has held its course for more than half a century, it may not be unreasonable for it to arrogate to itself the power to follow its own judgment in the matter of advertising remedies without ulterior aid in the selection of what appears to it to be acceptable. Especially it is disinclined to seek counsel and advice from the Sanhedrim of the A. M. A., if for no other reason than that for many years the *Journal* has studiously ignored the existence of the *Journal-Record* and during all that time has mentioned it only once and that in a highly derogatory connection. This attitude of the mouthpiece of the Council does not dispose us very strongly to run to mother's knee to learn if this or that will hurt us if we take it.

There is probably much good to be got from systematic analysis of proprietaries in the way of sifting the chaff from the wheat, but not a small proportion of the busy Council's painfully gathered information seems scarcely worth while. If a proprietary has merit, it will endure; if not, it will soon be found out and disappear. The medical profession may be very foolish, credulous and gullible, but it is not so foolish as to continue to use a remedy or combination that is mendacious in its claims and worthless as to results.

We have no quarrel with the Council and its mission, but we do object decidedly to the disposition of considering its findings in the light of a detection of moral obliquity and ethical delinquency on the part of physicians who prescribe and journals which advertise those proprietaries which have not received its imprimatur, and we also object to the employment of its investigations as a club to break the head of the independent medical journal. The independent medical journal ultimately may be put out of business if it regards as mandatory the admonitions of the Council and accepts the reprint as the book of the law. If it merely parallels the dependent medical journal, it is soon forspent and lags superfluous. Being good is largely a matter of convenience. The *Journal,* entrenched behind its impregnable subscription list, could live on the advertising usufruct of such unequivocally ethical preparations as castor oil and blue mass and in addition support a few people. But if the independent medical journal is going to meet its death, we know of one which, like Arnold von Winkelried, is going to draw as many spears to its breast as possible before the fall. Before this direful events occurs, may there not be an intrepid spirit here and there who will brave the Jovian wrath and invite those who are self-constituted censors and moral reformers to seek that sunless marsh where moral obliquity and ethical deliquency are said to reign supreme.—*Editorial, Atlanta Journal-Record of Medicine.*

---

Dr. D. L. Peeples, of Navasota, Texas, Surgeon Major in the Texas National Guard, has been pronounced insane and sent to the asylum at Austin.

